# Quantifying the Detection Sensitivity and Precision of qPCR and ddPCR Mechanisms for eDNA Samples

**DOI:** 10.1002/ece3.70678

**Published:** 2024-12-11

**Authors:** Gledis Guri, Jessica Louise Ray, Andrew Olaf Shelton, Ryan P. Kelly, Kim Præbel, Elizabeth Andruszkiewicz Allan, Nigel Yoccoz, Torild Johansen, Owen S. Wangensteen, Tanja Hanebrekke, Jon‐Ivar Westgaard

**Affiliations:** ^1^ Norwegian Institute of Marine Research Framsenteret Tromsø Norway; ^2^ Norwegian College of Fishery Science UiT the Arctic University of Norway Tromsø Norway; ^3^ NORCE Norwegian Research Centre AS, Climate & Environment Department Uni Research Environment Bergen Norway; ^4^ Northwest Fisheries Science Center NOAA Fisheries Seattle Washington USA; ^5^ School of Marine and Environmental Affairs University of Washington Seattle Washington USA; ^6^ Department of Arctic and Marine Biology UiT the Arctic University of Norway Tromsø Norway; ^7^ Department of Evolutionary Biology, Ecology & Environmental Sciences Universitat de Barcelona Barcelona Spain

**Keywords:** ddPCR, eDNA, fish monitoring, qPCR, quantification precision, sensitivity

## Abstract

Environmental DNA (eDNA) detection employing quantitative PCR (qPCR) and droplet digital PCR (ddPCR) offers a non‐invasive and efficient approach for monitoring aquatic organisms. Accurate and sensitive quantification of eDNA is crucial for tracking rare and invasive species and understanding the biodiversity abundance and distribution of aquatic organisms. This study compares the sensitivity and quantification precision of qPCR and ddPCR for eDNA surveys through Bayesian inference using latent parameters from both known concentration (standards) and environmental samples across three teleost fish species assays. The results show that ddPCR offers higher sensitivity and quantification precision, particularly at low DNA concentrations (< 1 copy/μL reaction), than qPCR. These findings highlight the superior performance of ddPCR for eDNA detection at low concentrations, guiding researchers towards more reliable methods for effective species monitoring. Additionally, this study indicates that a two‐step (detection and concentration) model increased the precision of qPCR results, useful for enhancing the robustness of eDNA quantification. Furthermore, we investigated the lower limit of quantification for ddPCR, providing insights on how such limit can be extended, which could also be applied to qPCR.

## Graphical abstract

This study compares the sensitivity and quantification precision of qPCR and ddPCR for eDNA surveys through Bayesian inference using latent parameters from both known concentration (standards) and environmental samples across three teleost fish species assays. The results show that ddPCR offers higher sensitivity and quantification precision, particularly at low DNA concentrations (< 1 copy/μL), than qPCR. These findings highlight the superior performance of ddPCR for eDNA detection at low concentrations, guiding researchers towards more reliable methods for effective species monitoring.

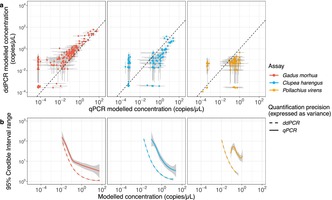

## 1 Introduction

Environmental DNA (eDNA)—DNA that is released into the environment by living organisms through various means, such as skin shedding, faeces, urine, and mucus secretion—has emerged as a powerful tool for detecting and monitoring aquatic organisms (Taberlet et al. 2012). The detection of eDNA in water or soil samples can provide a non‐invasive and efficient way to detect the presence of aquatic organisms (Wood et al. 2021), including rare, cryptic, or invasive species (Keller et al. 2022). Accurate quantification of eDNA is essential for reliable interpretation of ecological data such as population monitoring (Guri et al. 2024; Shelton et al. 2022), biodiversity assessment and species detection for invasive species monitoring (Sassoubre et al. 2016). The two most common methods used for quantification of DNA are quantitative PCR (qPCR) and droplet digital PCR (ddPCR). These quantitation techniques have diverse applications such as molecular biology (Taylor, Laperriere, and Germain 2017), epidemiology (Schneider et al. 2016) and environmental DNA studies (Dimond et al. 2022). In molecular biology and epidemiology, qPCR and ddPCR can be used to detect and quantify specific pathogens, aiding in the diagnosis and treatment of diseases. In environmental DNA studies, these techniques can assist in detecting biodiversity changes, tracking the spread of invasive species or monitoring the abundance of endangered species (Lodge et al. 2012). Here, we compare these two methods regarding (1) detection sensitivity (the probability of each method correctly identifying the presence of a target DNA) and (2) quantification precision (accuracy and consistency with which each method can quantify the concentration of DNA present in a sample) using laboratory standards and environmental samples in both methods.

Quantitative PCR (qPCR) with hydrolysis probes uses both template‐specific oligonucleotide primers and a fluorescently labelled oligonucleotide probe, all of which bind specifically to the DNA target. The amount of fluorescence that accumulates in real‐time during the PCR amplification process is proportional to the amount of DNA target present in the sample. During PCR, the cycle threshold value (Ct; cycle number at which the fluorescence signal generated by the probe in the PCR reaction reaches a threshold above background levels) is determined, providing an indirect measure of the amount of target DNA in the sample. For quantification of a DNA target from unknown samples, qPCR relies on parallel analysis of those unknown ironmental and standard samples with known concentrations of DNA fragments as reference points (Taylor et al. 2015) typically consisting of synthetic DNA fragments that match the target DNA sequence. A standard curve relating Ct values to incremental changes in DNA concentration is calculated, and quantification of unknown samples is achieved by extrapolating the amount of fluorescence emitted by the unknown samples (or indirectly measured as their Ct values) to the corresponding nominal concentration on the standard curve. qPCR is highly sensitive and specific but heavily dependent on the standard curves which are themselves subject to substantial technical variation due to pipetting error.

Droplet digital PCR (ddPCR) is a DNA quantitation technique that involves partitioning a sample into oil‐encapsulated nano droplets, each of which constitutes of independent PCR reaction. Partitioning aims to create a large population of nano droplets with a predictable distribution of droplets containing zero, one or several copies of the target DNA. Amplification of the target DNA fragment within each individual droplet during PCR results in fluorescence signal accumulation at the end of PCR, which can then be measured in all individual droplets using microfluidic droplet detection. Dependent upon the magnitude of fluorescence signal (measured at the end of the PCR), each droplet is then counted as either a positive or a negative detection event for the target DNA. The distribution of DNA molecules into the droplets follows a Poisson distribution, where the rate parameter (*λ*) represents the rate of which the number of droplets containing at least one copy of the target DNA. By applying Poisson statistics, ddPCR accurately quantifies the absolute number of target DNA molecules in the original sample based on the observed proportion of positive droplets (Hindson et al. 2011), thus making ddPCR absolute and independent of standard curves (Taylor et al. 2015). However, while ddPCR eliminates the need for standard curves in quantification, it still requires assay optimisation using standard samples to ensure accuracy unless prior optimisations have been conducted for the specific set of primers.

Environmental samples generally have very low target‐species eDNA concentrations, which can impair the detection and quantification of organism occupancy and abundance (Marques et al. 2024). Selecting a method with the highest sensitivity and precision is thus crucial to overcoming these challenges. Although results from both methods are strongly and linearly correlated (Campomenosi et al. 2016; Jerde et al. 2016; Nathan et al. 2014; Verhaegen et al. 2016), ddPCR has demonstrated higher sensitivity (detection probability) in clinical microbiology (Campomenosi et al. 2016; Taylor et al. 2015), environmental molecular ecology (Jerde et al. 2016) and rare species detection (Mauvisseau et al. 2019). Additionally, ddPCR has shown higher precision of concentration estimates compared to qPCR (Hindson et al. 2013; Taylor et al. 2015), although this improved precision has not been empirically quantified. Quantifying the extent and specific conditions under which ddPCR demonstrates improved precision can help researchers allocate resources more effectively based on their specific goals and required level of precision.

Any difference in precision between the two techniques has been attributed primarily to ddPCR being less susceptible to inhibition than qPCR (Mahendran et al. 2020; Mauvisseau et al. 2019). Several studies have attempted to circumvent inhibition of qPCR by modifying reaction chemistry. For example, Hindson et al. (2013) used ABI and Bio‐Rad Master Mix for qPCR, while Doi et al. (2015) used Environmental and Universal Master Mix containing AmpliTaq DNA polymerase variants that are less prone to inhibitors to improve sensitivity and concentration accuracy. Although substantial attention has been given to laboratory techniques and chemical solutions to enhance sensitivity and accuracy by these methods, particularly for qPCR, little emphasis has been placed on developing mathematical approaches to provide higher sensitivity and quantitative precision and accuracy.

The aim of this study is to empirically assess the sensitivity and quantification precision of qPCR and ddPCR quantification methods using improved concentration estimation formulas applied to eDNA samples. In the case of qPCR, we adopt the framework used in McCall et al. (2014) and Shelton et al. (2022); for ddPCR we adopt the model used in Guri et al. (2024), which in short refines quantity estimations via a model that makes use of the relationship between the binomial and Poisson distributions (Hindson et al. 2011). We present a Bayesian analysis designed to independently model DNA quantities for qPCR and ddPCR using samples with known DNA concentrations (standards, ranging from 10^−3^ to 10^6^ copies/μL, see Table S2) alongside environmental samples for three different teleost fish assays. We used the same set of standard and environmental samples on both methods (qPCR and ddPCR). In addition, this study aims to identify limitations or biases associated with new models designed for enhanced concentration estimation and to provide recommendations for optimising quantitative eDNA protocols in future studies.

## 2 Methods

### 2.1 eDNA Water Sampling, Filtration and Extraction

We surveyed a total of twelve distinct sampling locations over the span of 3 years in Balsfjord and eight locations over 2 years in Frakkfjord (in the north of Norway), on the R/V Kristine Bonnevie, as part of Norwegian coastal surveys. No sampling permission was required by local authorities. At every sampling station, we collected duplicates or triplicates (as biological replicates) each of 5 L of seawater from three depths, surface (10 m), the pycnocline (the layer of highest density, approximately 50 m deep) and the bottom layer (10 m above the seafloor). From 153 field samples, we filtered 2 L of seawater using 0.22 μm Sterivex filters (MerckMillipore) and a peristaltic pump (multi‐channel flow Heidolph Hei‐Flow Advantage 01), and afterwards, the filters were sealed into sterile 50 mL Falcon centrifuge tubes and stored at −20°C until transported to laboratory facilities and preserved at −80°C until extraction. Negative controls (11 water blanks and 4 air blanks) were taken across all stations to indicate potential contamination. We used the DNeasy PowerWater Sterivex Kit (Qiagen GmbH) to extract the eDNA (for more details on extraction see Guri et al. 2023). The same set of standard and environmental samples were used downstream in both methods (qPCR and ddPCR).

### 2.2 Standard Samples

The DNA from tissue samples of *Gadus morhua* (Atlantic cod, hereafter ‘cod’), *Clupea harengus* (Atlantic herring, hereafter ‘herring’) and *Pollachius virens* (saithe) was extracted using the DNeasy Blood & Tissue Kit (Qiagen GmbH, Hilden Germany). To create standard samples, we amplified the 103‐bp region of the ATPase and cytochrome b regions for cod and saithe, respectively, using specific primers (Table 1) with the thermocycler program: an initial 5 min at 95°C; followed by 40 cycles of 1 min at 95°C, 1 min at 62°C and 1 min at 72°C; and a final phase at 72°C for 10 min. The reactions were run on SimpliAmp Thermal Cycler machine (Thermo Fisher Scientific). We performed a total of 24 reactions (8 per species), each in 25 μL volume including 12.5 μL of TaqMan Environmental Master Mix 2.0, 1.25 μL of each of forward and reverse primers (10 nM concentration each), 8 μL of RNA‐free water and 2 μL of genomic DNA template for the above‐listed species. The PCR products were thereafter examined by gel electrophoresis, and replicate reactions with the expected product size were pooled for subsequent purification, wherein primer‐dimers were eliminated using the MinElute PCR Purification Kit (Qiagen GmbH, Hilden Germany). The DNA concentration in the pooled samples was measured using Qubit quantification system using the High Sensitivity dsDNA assay. The samples were converted from mass per volume (ng/μL) to copies per volume (copies/μL) using the fragment size of each marker. Subsequently, samples underwent a series of 10‐fold dilutions to achieve final concentrations ranging from 10^−3^ to 10^6^ copies/μL (10^−3^–10^4^ used in ddPCR and 10^−1^–10^6^ used in qPCR).

**TABLE 1:** Sequences for qPCR and ddPCR assays targeting 103‐bp region of the ATPase gene of Atlantic cod ( *Gadus morhua* ) and saithe ( *Pollachius virens* ), and cytochrome b sequence of Atlantic herring ( *Clupea harengus* ). All gene regions belong to the mitochondrial DNA.

Target	Primers		Sequence		Dye
*Gadus morhua*	Forward	GAD‐FII	GCAATCGAGTYGTATCYCTHCAAGGAT	(Taylor et al. 2002) (Nash et al. 2012)	
	Reverse	GAD‐R III	GCAAGWAGYGGHGCRCADTTGTG		
	qPCR probe	Custom	CTTTTTACCTCTAAATGTGGGAGG		FAM
	ddPCR probe	Custom	CTTTTTACCTCTAAATGTGGGAGG		VIC
*Clupea harengus*	Forward	Cluhar_CYBF14928	CCCATTTGTGATTGCAGGGG	(Knudsen et al. 2019) (Knudsen et al. 2019)	
	Reverse	Cluhar_CYBR15013	CTGAGTTAAGTCCTGCCGGG		
	qPCR probe	Cluhar_CYBP14949	TACTATTCTCCACCTTCTGTTCCTC		JOE
	ddPCR probe	Cluhar_CYBP14949	TACTATTCTCCACCTTCTGTTCCTC		FAM
*Pollachius virens*	Forward	Saithe‐F	GAATCCCAATAATTTTAATAGCCT	Unpublished	
	Reverse	Saithe‐R	TCGATTGCTTAGTCATCGAGA	Unpublished	
	qPCR probe	Custom	TGATTACTCATCCCTACG		Cy3
	ddPCR probe	Custom	TGATTACTCATCCCTACG		FAM

### 2.3 qPCR

Prior to running all the qPCR samples (environmental *n* = 168 and standard samples *n* = 16, i.e., 8 per each multiplexing assay), assay optimisation was performed to achieve satisfactory amplification efficiency (Figure S1). All qPCR environmental samples were analysed in triplicates (and some in quadruplicates when enough DNA template was available; see Table S2 for standard samples number of technical replicates) on an Applied Biosystems 7500 Fast real‐time PCR System machine (Thermo Fisher Scientific) using the thermocycler protocol of 10 min at 95°C for denaturation followed by the cycling stage of 52 cycles (the first and second plates were run on 42 and 45 cycles due to optimisation issues) of 15 s at 95°C and 1 min at 58°C. Cod assays were duplexed with either herring or saithe assays for samples in Balsfjord or Frakkfjord, respectively. 5′‐hydrolysis probes were labelled with 6‐FAM for cod detection, JOE for herring detection or Cy3 for saithe detection (Table 1), and all probes were modified at their 3′‐end with the quencher moiety BHQ1. All thermocycler reactions were run in 20 μL volume consisting of 10 μL of TaqMan Environmental Master Mix 2.0, 1 μL of each primer (forward and reverse, 10 nM concentration each; see Table 1), 0.5 μL of probe (10 nM concentration), 3 μL of dH_2_0 and 2 μL of DNA template. Negative controls were run together with the samples. Duplexed standard dilution series containing 10^−1^–10^6^ copies μL^−1^ of purified target fragments were included in all qPCR plates to generate standard curves and verify performance consistency between qPCR runs.

### 2.4 ddPCR

Prior to running all the ddPCR samples (environmental *n* = 168 and standard samples *n* = 16, i.e., 8 per each multiplexing assay), ddPCR assays were optimised by testing different primer/probe concentrations and annealing/elongation temperature gradients to identify the conditions that resulted in the highest fluorescence difference between positive and negative droplets. All ddPCR environmental samples were analysed in triplicates (and some in quadruplicates when enough DNA template was available; see Table S2 for standard samples number of technical replicates). Herring (6‐FAM) or saithe (6‐FAM) assays were run in duplexed reactions with the cod (VIC) assay on samples from Balsfjord or Frakkfjord, respectively. Duplex cod/saithe ddPCR reactions consisted of 11 μL of ddPCR Supermix with no dUTP (Bio‐Rad), 11.9 pmol of each primer, 3.5 pmol of probe, 0.04 μL of RNA‐free water and 5.5 μL of DNA template. For duplex cod/herring assays we used the same volumes of Supermix, DNA template and cod primers and probe, and for herring we used 4.4 pmol of forward primer, 1.32 pmol of reverse primer, 4.4 pmol of probe and 2.35 μL of RNA‐free water. The total volume for all ddPCR reactions was 22 μL, from which 20 μL were pipetted into the reaction well to ensure volume precision. Droplets were generated according to manufacturer instructions, aiming for 20,000 droplets per reaction. Emulsion PCR was performed in a C1000 Touch Thermal Cycler with 96‐Deep Well Reaction Module (Bio‐Rad) with a program of 10 min at 95°C, 44 cycles for 1 min at 95°C and 2 min at 55.6°C, with a ramp rate of 2°C per s, followed by 10 min at 98°C and stored at 4°C. Room temperature‐equilibrated ddPCR plates were then analysed using a Q×200 droplet reader (Bio‐Rad). Additional runs consisted of duplexed standard samples of nominal concentration ranging from 10^−3^ to 10^4^ copies/μL using the same protocol and amplification program.

### 2.5 Bayesian Approach and Statistics

#### 2.5.1 Detection Probability (i.e., Sensitivity) Between Methods

We established the relationship between the starting DNA concentration and the probability of positive target detection for both methods (qPCR and ddPCR) independently using standard samples of known concentration by using the logistic regression model:

(1) $$Z_{\mathrm{ijk}}∼\text{Bernoulli}(\theta_{\mathrm{ij}})$$

(2) $$\text{logit}\left(\theta_{\mathrm{ij}}\right)=\varphi 0_{i}+\varphi 1_{i}\times \log_{10}C_{\mathrm{ij}}$$

where *Z* is the binary outcome for sample (*j*) and technical replicate (*k*) of a target (*i*) being present or absent following a Bernoulli distribution given the probability of detection *θ*. The parameters *φ*0 and *φ*1 are the intercept and the slope respectively in the logistic function with nominal DNA concentration in copies/μL (C) as the predictor variable. Subsequently, we compared the differences in probabilities of detection (*θ*_*i*_) between the two methods across the range of tested concentrations.

#### 2.5.2 Precision Estimation Between Methods

To estimate qPCR‐modelled concentrations, we employed the approach formulated by McCall et al. (2014) and Shelton et al. (2022). In short, to estimate the starting DNA concentration, the model jointly combines the use of a detection probability model (based on the technical replicate of samples, Equations (1) and (2)) and the continuous model as follows:

(3) $$Y_{\mathrm{ijk}}∼\text{Normal}(\mu_{\mathrm{ij}},\sigma_{\mathrm{ij}})\text{ if }Z_{\mathrm{ijk}}=1$$

(4) $$\mu_{\mathrm{ij}}=\beta 0_{i}+\beta 1_{i}\times \log_{10}C_{\mathrm{ij}}$$

(5) $$\sigma_{\mathrm{ij}}=e^{\left(\gamma 0_{i}+\gamma 1_{i}\times \log_{10}C_{\mathrm{ij}}\right)}$$

where *Y*, the observed cycle threshold (Ct), follows a normal distribution with mean *μ* (mean Ct) with some standard deviation σ (Equation 3). We model μ as a linear function of starting eDNA concentration (*C*) with intercept and slope *β*0 and *β*1 (Equation 4). The standard deviation of the observed *Y* is an exponential function of starting eDNA concentration with intercept and slope *γ*0 and *γ*1 (Equation 5).

To estimate ddPCR‐modelled concentration, we used Poisson statistics (Hindson et al. 2013; Equation 6) in the form of cloglog transformation (Equation 7) as follows:

(6) $$C=-\ln\left(1-\omega \right)\times V^{-1}$$

(7) $$\ln\left(C\right)=\text{cloglog}\left(\omega \right)-\ln\left(V\right)$$

where ω is the proportion of positive droplets, and V is the ddPCR oil droplet volume. We assume that the number of positive droplets (W) follows a binomial distribution with probability of success (*ω*) given the total number of droplets generated (U; Equation 8). Following Equation (7) we establish a linear relationship between ω and starting DNA concentration (C) using an intercept and a slope κ0 and κ1 as follows:

(8) $$W_{\mathrm{ijk}}∼\text{Binomial}(\omega_{\mathrm{ij}},U_{\mathrm{ijk}})$$

(9) $$\text{cloglog}\left(\omega_{\mathrm{ij}}\right)=\kappa 0_{i}+\kappa 1_{i}\times \log_{10}C_{\mathrm{ij}}$$

By extrapolation, following Equations (7) and (9), *κ*0 and *κ*1 should equal ln(V) and $\frac{\ln\left(C\right)}{\log_{10}\left(C\right)}$ respectively for assays with efficiency of 100%. In contrast to Equation (7) (the default ddPCR software formula), in our formulation (Equation 9), the ratio of positive droplets of all technical replicates (*k*) of a sample (*j*) are aggregated (sharing information for a sample (*j*)), hence minimising the weight of individual stochastic observations. In principle and in both models (qPCR and ddPCR), the known concentration from standards informs the intercept and the slope parameters which informs the initial concentration of unknown samples (*C*). This happens simultaneously by maximising the posterior probability through Bayesian inference using the Hamiltonian MCMC algorithm.

The two parts of the qPCR model (Equations (1), (2), (3), (4), (5)) were run jointly, but independently, from the ddPCR model (Equations 8 and 9); thus the initial DNA concentration, despite denoted C for both models for simplicity, is not estimated jointly between the two models. To assess the robustness of the methods studied, we calculated and compared the uncertainty of each model for eDNA quantification. We determined the uncertainty (quantification precision) as the difference between the 2.5% and 97.5% credible interval range of the modelled concentration (C) for qPCR and ddPCR; thus higher uncertainty (difference of the credible interval range) indicates lower precision and vice‐versa.

Furthermore, we explored the potential benefits of incorporating the detection probability model (Equations 1 and 2) alongside the continuous model (Equations (3), (4), (5)) for improving the quantification precision and accuracy (the latter is not estimated in this article) of qPCR. To achieve this, we ran the continuous model independently and in conjunction with the detection probability model, measuring the variance in each scenario. Subsequently, we compared the precision (95% CI) to assess the enhanced value introduced by the detection probability model.

## 3 Results

### 3.1 Sensitivity of Methods

**FIGURE 1:**
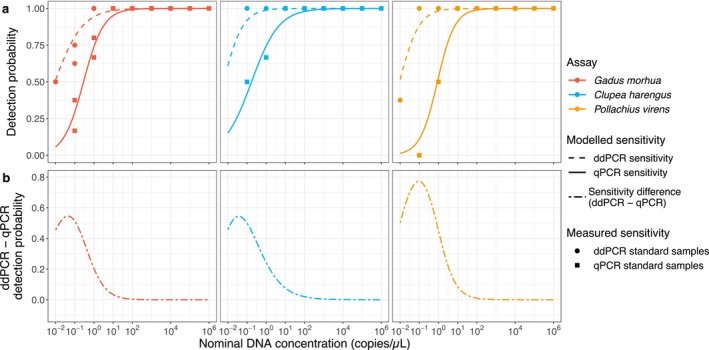
Sensitivity of (a) ddPCR and qPCR for three assays (cod = red, herring = blue, and saithe = orange) shown as modelled detection probability as a function of nominal DNA concentration. Difference of detection probability (b) between ddPCR and qPCR is also shown over the eDNA concentration where positive values indicate higher ddPCR detection probability and zero indicates similar detection probabilities between the two employed methods.

All the DNA concentrations hereafter are expressed per 20 μL reaction volume, thus 1 DNA copy in a 20 μL reaction = 0.05 copies/μL reaction volume (ca. 10^−1.3^ copies/μL). Standard samples of herring with concentration 10^−2^ showed reasonably higher concentrations and were therefore removed from the downstream analysis. The sensitivity for ddPCR standard samples showed on average a 0.5 detection probability at 10^−2^ copies/μL for all assays (Figure 1a). Conversely, the qPCR results indicated a lower probability of detection compared to ddPCR. Specifically, for the cod and herring assays, the 0.5 detection probability was observed at a concentration of ca. 10^−0.7^ copies/μL, while for the saithe assay, it was indicated at 1 copies/μL for qPCR assays (Figure 1a). ddPCR had a higher probability of detection for concentrations ranging from 10^−2^ to 10^0^ copies/μL for all target species (Figure 1b). Sensibly, the advantage of ddPCR for detection varied by assay: cod and herring assay of ddPCR showed 3 times higher detection probability (0.75 and 0.25 detection probability for ddPCR and qPCR respectively) at DNA concentrations of 10^−1^, while for the same concentration, saithe showed 8 times higher detection probability (0.88 and 0.11 detection probability for ddPCR and qPCR, respectively) when compared to qPCR (Figure 1b).

### 3.2 Quantification Precision of Methods

**FIGURE 2:**
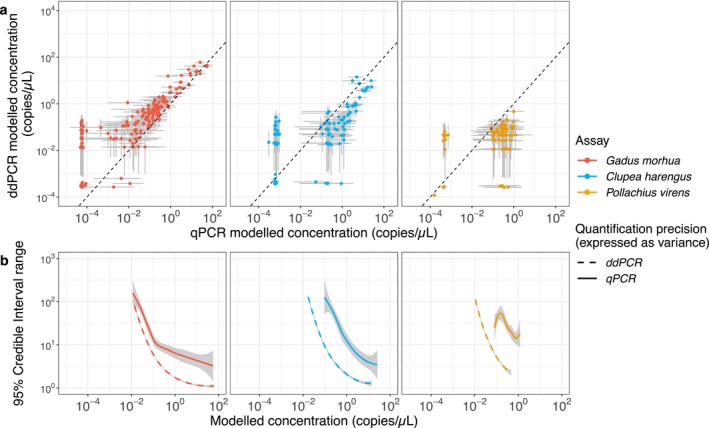
Comparison of modelled quantities between qPCR and ddPCR and their credible interval (grey bars) for three assays (cod = red, herring = blue, and saithe = orange; a). Concentration below 10^−3^ for both methods are considered non‐detect. The difference between ddPCR and qPCR credible intervals is also shown (b) and grey shade for its variance.

qPCR and ddPCR model estimates of starting DNA concentration in environmental samples were positively correlated (Figure 2a). The qPCR model slightly underestimated the starting DNA concentration (C from Equations 2 and 4) for cod detection relative to ddPCR model (C from Equation 9), while it overestimated starting DNA concentrations for herring and saithe detection. Consistent with the sensitivity analysis above, ddPCR yielded fewer negative detections from environmental samples (*n* = 38) compared to qPCR (*n* = 86). Both methods estimated fewer than 10^−3^ target copies/μL for all negative samples. Furthermore, the qPCR model exhibited a wider credible interval range (lower precision for estimating starting DNA concentration) for all assays and template concentrations (Figure 2b). The quantitation variability (qPCR and ddPCR) was inversely correlated with DNA concentration, indicating a positive relationship between precision and target concentration. For the cod assay in particular, the variability was one order of magnitude, while for herring and saithe, it was approximately two orders of magnitude (see the difference between dashed line and dotted line in Figure 2b).

The modelled cod and saithe ddPCR parameters (*κ*0 = −7.07 and *κ*1 = 2.3 from Equation (9), Table S1) mirrored the default parameters of ddPCR (default Poisson statistics in QuantaSoft software Equation (7); Figure S3). Conversely, the modelled parameters for herring detection were higher than the aforementioned assays (*κ*0 = −7.54 and *κ*1 = 2.24, Table S1).

### 3.3 Two‐Step Model of qPCR Quantification Precision

**FIGURE 3:**
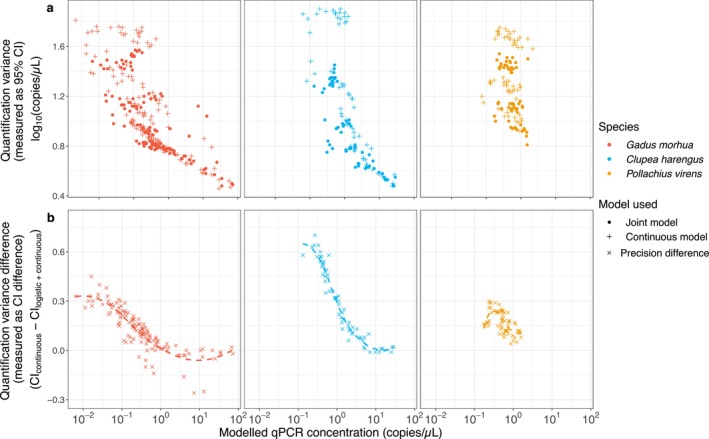
Quantitation precision (expressed as the 95% CI quantification variance) of continuous model (plus symbols) and two‐step model (continuous and detection probability model for qPCR; dots symbols) for three assays (cod = red, herring = blue, and saithe = orange; a) and their difference in precision (b; crosses symbol). Positive values in panel (b) reflect wider CIs for the continuous model alone and hence identify areas of parameter space in which the two‐step model helps to increase precision.

We estimated the marginal value of the two‐step (detection probability + continuous) qPCR model with the simpler continuous model by comparing the uncertainty of estimated concentrations for each. The quantification precision, expressed as variance, for continuous model alone was lower (i.e., higher variance) than the precision under the two‐step model (Figure 3). We observed an increase in quantification precision by an average of 0.5 orders of magnitude for samples with starting DNA concentrations lower than 10^2^ copies/μL (Figure 3b). Note that, as sample concentrations increase beyond approximately 10 copies/μL, the precision of the two qPCR models becomes equivalent.

### 3.4 Lower and Upper Limit of Detection and Quantification

Both quantitation methods have estimable lower limits of detection and quantification, which, in case of qPCR has been previously reported as LoD and LoQ (Forootan et al. 2017; Klymus et al. 2020) by using the coefficient of variation (CV) and probability of detection as thresholds (Figure S5). However, applying LoD and LoQ for qPCR in this study would not be appropriate due to the fundamental differences between Bayesian inference and the frequentist approach. Instead, we propose an alternative method for estimating the lower limit of detection and quantification (hereafter referred to as C_low‐threshold_, denoted as C_lt_), defined by the presence of a single DNA copy in the reaction volume (20 μL in this study). Assuming that the assay efficiency and the qPCR machine sensitivity are 100% (i.e., if present, it is detectable and quantifiable) $C_{\mathrm{lt}}=\frac{1\;\text{copy}}{20\;\mathrm{\mu L}}=\frac{10^{-1.3}\text{copies}}{\mathrm{\mu L}}$. Similarly, for ddPCR, *C*_*lt*_ can be defined as the presence one single positive droplet from the pool of generated droplets (U). The lower limit of detection and quantification (*C*_*lt*_) for ddPCR mechanism can be calculated in the following equation derived from Equation (7):

(10) $$\ln\left(C_{\mathrm{lt}}\right)=\text{cloglog}\left(1/U\right)-\ln\left(V\right)$$

**FIGURE 4:**
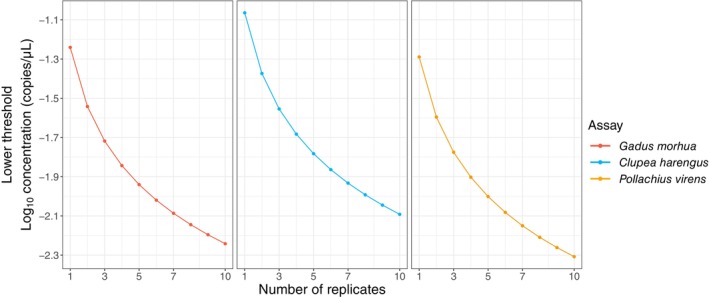
The lower limit of ddPCR detection and quantitation (20 μL reaction volume and ca. 20,000 droplets generated) as a function of number of replicates for three assays (cod = red, herring = blue, and saithe = orange) estimated using Equation (9).

Given that many of the parameters involved in ddPCR workflow are constrained, the most effective way to decrease the *C*_*lt*_ is by increasing the number of droplets generated and analysed. Since each droplet in ddPCR acts as an independent end‐point PCR reaction, increasing the number of ‘amplification replicates’ of the sample by increasing the number of droplets expands the ranges of lower limits of detection and quantification (Figure 4). In a similar manner for qPCR, increasing the number of technical replicates can further reduce the detection and quantification limits resulting in very similar *C*_*lt*_ as those of ddPCR (Figure 4). Differently from qPCR, ddPCR has an upper limit of detection and quantification (*C*_*ut*_) which can be defined by the following equation:

(11) $$\ln\left(C_{\mathrm{ut}}\right)=\text{cloglog}\left(\left(1,-,U\right)/U\right)-\ln\left(V\right)$$

## 4 Discussion

Sensitive and precise quantification of eDNA is essential for understanding the presence, distribution and abundance of aquatic organisms and for informing management decisions related to biodiversity conservation, invasive species control and ecosystem health. Estimating the sensitivity and precision of quantitation methods can provide guidance to researchers in choosing the most effective techniques for quantifying organisms of interest. The current study estimated and compared the sensitivity and precision of qPCR and ddPCR for DNA quantitation using three detection assays for important teleost fish species. This study showed that ddPCR outperformed qPCR by showing higher sensitivity and precision, especially at low DNA concentration (10^−2^–10^0^ copies/μL), typical of environmental DNA concentrations. To our knowledge this study is the first of its kind to empirically measure the advantages of ddPCR quantitation methods over those of qPCR along a series of DNA concentrations.

### 4.1 Comparison Between qPCR and ddPCR (i.e., Sensitivity and Quantification Precision)

This study represents a comparison of qPCR and ddPCR detection platforms with regard to empirical and modelled performance indicators. While our findings align with those of prior research, we independently quantify the sensitivity and the quantification precision of these methods through empirical measurements. Although the results found in this study are consistent with previous findings, here we empirically measure methods' sensitivity and quantification precision. One study concluded that qPCR detection loses precision at starting DNA concentration lower than 1 (10^0^) copies/μL (Mahendran et al. 2020). Concentrations lower than this lead to an increased CV that surpasses the limit of detection for qPCR, thus introducing the risk of false negative detections (Hindson et al. 2013; McCall et al. 2014). Such threshold is indicated to be a general eDNA threshold stochastic detection for qPCR.

By contrast, we found that our results provide empirical evidence that ddPCR has higher sensitivity than qPCR for concentrations lower than 10^0^ copies/μL. This may be attributed to the fact that ddPCR is based on end‐point detection, which circumvents issues related to PCR inhibition (untested in this article) and variation in amplification efficiencies prior to reaching amplification plateaus (Taylor et al. 2015; Yang et al. 2014). Verhaegen et al. (2016) have shown that ddPCR and qPCR (when using Environmental Master Mix) are not affected by the PCR inhibition (induced as bile salt). However, their standard sample target concentration was relatively high (10^3^ copies/μL) and thus may not accurately reflect patterns at the low concentration (10^−2^–10^0^ copies/μL) which are often observed in eDNA‐based surveys.

Despite higher ddPCR detection sensitivity at low target concentrations, we observed a positive correlation between starting DNA concentration estimates for qPCR and ddPCR, in agreement with findings from previous studies (Campomenosi et al. 2016; Doi et al. 2015; Jerde et al. 2016; Nathan et al. 2014; Tang et al. 2016). While we did notice slight differences between assays, both approaches provided similar overall estimates of concentration. However, when considering the precision of each method, we found that ddPCR outperformed qPCR in estimating quantities by half to one order of magnitude across all concentrations. This may again be attributed to digital resolution being higher or the endpoint nature of ddPCR, which avoids the stochastic variation that can occur between cycles of DNA amplification. One of the explanations for low detectability and higher variance of qPCR could be the low number of DNA molecules used in such reactions (2 μL instead of 5 μL used in ddPCR), although Takahara, Minamoto, and Doi (2015) instead concluded from their experiments that using 2 μL of DNA template showed higher detection rate due to less PCR inhibition (Doi et al. 2015; Tang et al. 2016). As eDNA extracts vary greatly in content of PCR inhibitors, it is difficult to make general statements about template volume and potential negative impact on PCR amplification efficiency. In the present study, the extent of observed platform‐dependent variance depended on the assay in question, with the cod assay showing lower variation between the two methods compared to the herring assay.

### 4.2 ddPCR (Non)reliance External Calibration

Although one advantage of ddPCR is freedom from the standard curves (typically required for qPCR; Verhaegen et al. 2016), our ddPCR results indicate an assay‐specific difference in the relationship between positive droplets and nominal concentrations (Figure S3) and therefore the value of interrogating samples of known concentration with ddPCR. We can assume from Equations (7) and (9) that *κ*0 = ln(V) and *κ*1 = ln(10). Given our estimates for the latent parameters (Table S1) we conclude that a single droplet volume for cod and saithe assay (in concordance with that provided by BioRad) is ~0.00085 μL. Parameters of herring assay indicated a droplet volume 1.6 times smaller than those of cod and saithe (~ 0.00054 μL). Such values would, by extrapolation, indicate that fewer DNA molecules are being encapsulated into a single droplet, or the droplets generated for herring detection were smaller. However, droplet metrics indicated similar numbers of accepted (positive + negative) droplets for all three assays (data not shown), which challenges the possibility that herring droplets were smaller (as all samples were run in 20 μL reactions). Additionally, we noticed that the fluorescence amplitude for herring was significantly higher compared to the other two assays (Figure S4) yet unable to explain how that can affect our parameters. Subsequently, DNA degradation of herring standard samples (see the difference between nominal and measured herring standard concentration in Figure S2) could potentially be the main explanation for the difference between nominal and known concentrations, subsequently altering the *κ*0 parameters (ln(V); Figure S3). Moreover, *κ*1 could be used to assess assay efficiency. We suggest that standard curve inclusion during ddPCR assay optimisation may be a useful supportive tool to increase understanding of ddPCR assay behaviour and allow targeted calibration measures to improve application precision and accuracy.

Our analysis suggested that the default calculations in the QuantaSoft ddPCR software tend to overestimate low DNA concentrations from standard samples (Figure S2). We denote that in scenarios when quantitation accuracy is paramount, the inclusion of standard samples and technical replicates information sharing (as done in this model) may become instrumental for attaining the necessary level of accuracy. This can be done using the models described here or substituting *κ*1 with *x*log(*x*) function (non‐linear function as x→0 but approximates *x* (linear function) as *x* > 0) in Equation (9) which can additionally account for the stochastic detection at low concentration. Conversely, in situations where accuracy at concentration below 10^−2^ copies/μL is not the primary focus of the study, resorting to the model described here remains an acceptable alternative approach and default ddPCR software calculations for accuracy at concentration higher than 10^−1^ copies/μL. It is essential to recognise that, in the latter case, any under and overestimation by ddPCR will be uniform across all samples.

## 5 Conclusion

This study provides valuable insights into the strengths and limitations of qPCR and ddPCR methods and helps to inform best practices for eDNA research and monitoring in the future. We find that ddPCR has higher sensitivity and precision at low DNA concentrations, which are of particular relevance for eDNA‐based detection surveys. We present empirical data suggesting that for eDNA concentrations less than 1 copy/μL it is advantageous to switch to ddPCR quantitation method. Furthermore, we recommend the use of standard samples when optimising ddPCR assays as it increases understanding of assay technical performance and facilitates troubleshooting when assay efficiency is unsatisfactory. We show that the implementation of a qPCR detection probability model, when used jointly with the continuous model (two‐step model), improves quantification precision. We strongly encourage the use of technical replicates not only as a tool to increase the precision and accuracy of measurement but also as means to reduce statistical uncertainty of detection at low target concentrations.

## Author Contributions

**Gledis Guri:** conceptualization (equal), data curation (lead), formal analysis (lead), methodology (lead), validation (equal), visualization (lead), writing – original draft (lead), writing – review and editing (lead). **Jessica Louise Ray:** conceptualization (equal), funding acquisition (equal), methodology (equal), resources (equal), supervision (equal), visualization (equal), writing – review and editing (equal). **Andrew Olaf Shelton:** conceptualization (equal), formal analysis (equal), supervision (equal), validation (equal), writing – review and editing (equal). **Ryan P. Kelly:** conceptualization (equal), formal analysis (supporting), resources (equal), supervision (equal), validation (equal), writing – review and editing (equal). **Kim Præbel:** conceptualization (equal), methodology (equal), resources (equal), supervision (equal), validation (equal), writing – review and editing (equal). **Elizabeth Andruszkiewicz Allan:** conceptualization (equal), formal analysis (equal), supervision (equal), validation (equal), visualization (equal), writing – review and editing (equal). **Nigel Yoccoz:** conceptualization (equal), formal analysis (equal), supervision (equal), validation (equal), writing – review and editing (equal). **Torild Johansen:** conceptualization (equal), funding acquisition (lead), project administration (lead), resources (equal), supervision (lead), writing – review and editing (equal). **Owen S. Wangensteen:** conceptualization (equal), methodology (equal), project administration (equal), resources (equal), supervision (equal). **Tanja Hanebrekke:** conceptualization (equal), data curation (equal), methodology (equal). **Jon‐Ivar Westgaard:** conceptualization (equal), data curation (equal), formal analysis (equal), methodology (equal), project administration (equal), resources (equal), supervision (equal), writing – review and editing (equal).

## Conflicts of Interest

The authors declare no conflicts of interest.

## Supporting information


Data S1.

## Data Availability

The data and the model's script used in this article are available in Zenodo repository. (Dataset reference DOI: 10.5281/zenodo.10575343).
