# Supplementary material for: Quantifying the Detection Sensitivity and Precision of qPCR and ddPCR Mechanisms for eDNA Samples
Source: Ecol Evol. 2024 Dec 11;14(12):e70678. doi: 10.1002/ece3.70678 (PMC11634988; doi:10.1002/ece3.70678)
Supplement: Supplementary file 1 — Data S1. [file ECE3-14-e70678-s001.pdf]

Supplementary material

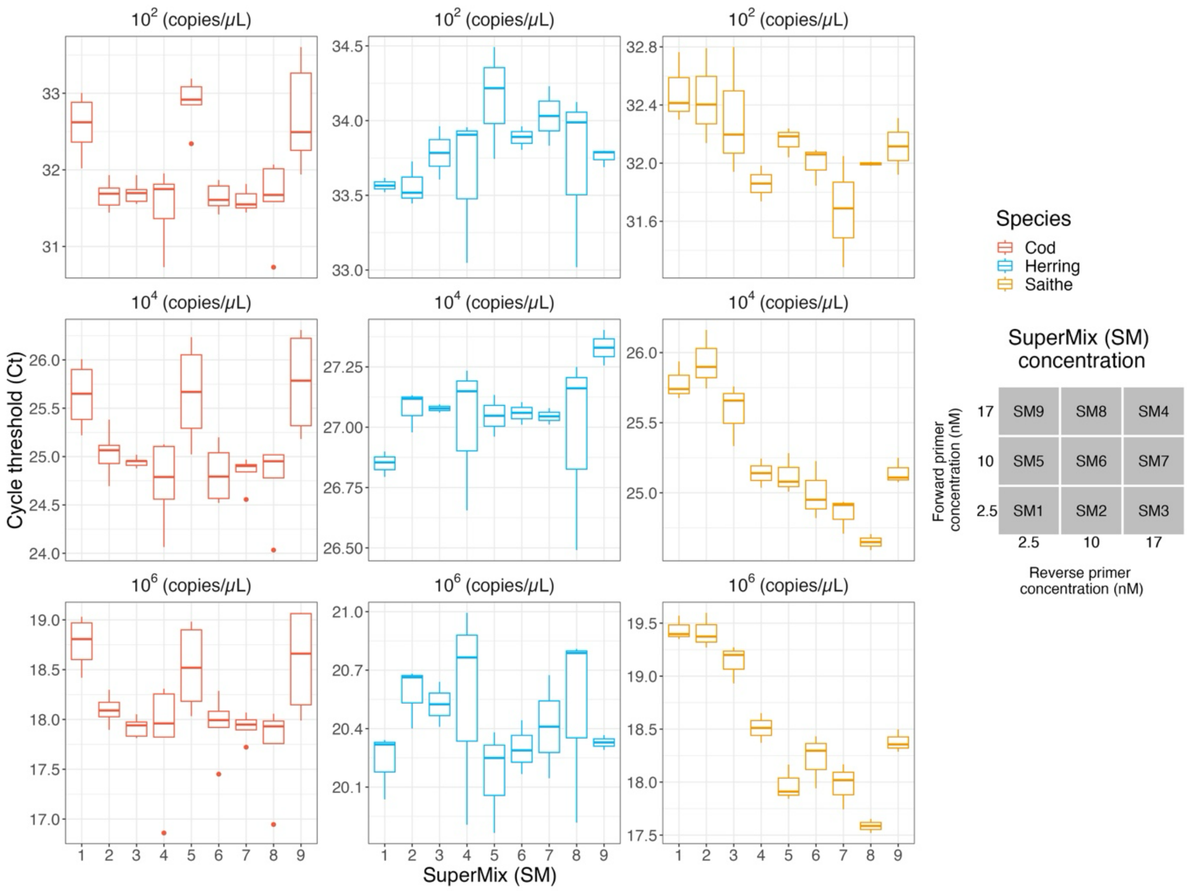

Figure S1. Optimisation runs for all three assays (cod = red, herring = blue, and saithe = orange) by using 9 different mixtures of Forward and Reverse primers (SM 1 to 9) and three nominal concentrations (10^2, 10^4, and 10^6 copies/μL). Lowest Ct indicates the highest efficiency.

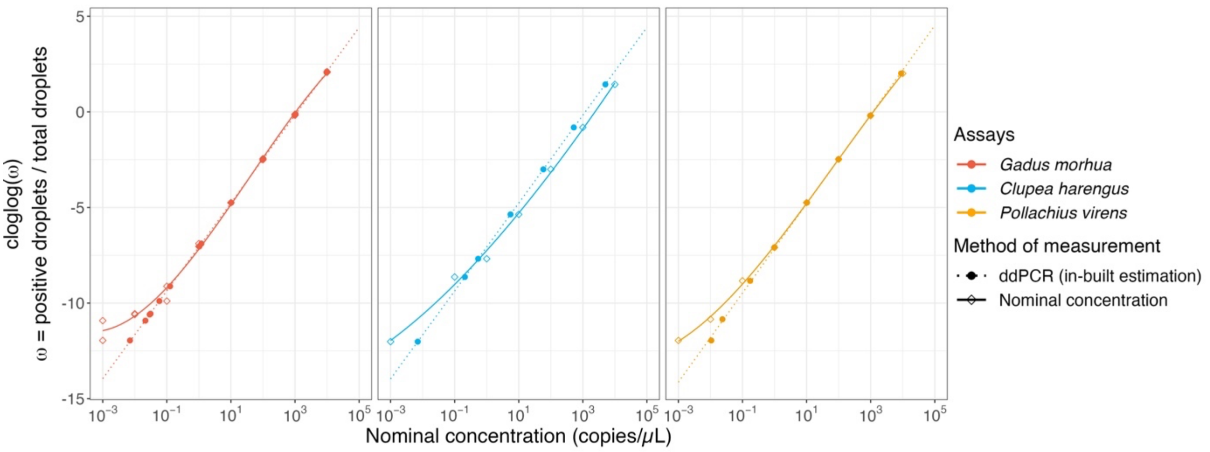

Figure S2. The relationship between proportion of positive droplets and nominal DNA concentration (line) and ddPCR default software estimation (dotted line) for three assays (cod = red, herring = blue, and saithe = orange) using ddPCR mechanism of measurement.

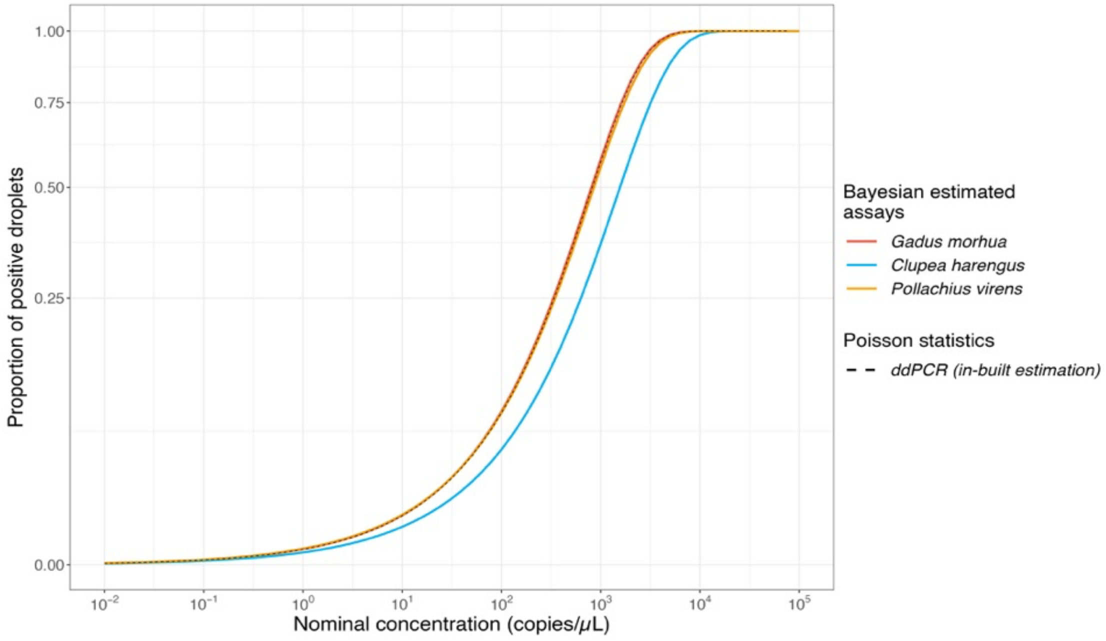

Figure S3. The relationship between proportion of positive droplets over the total number of droplets and nominal eDNA concentrations for three assays (cod = red, herring = blue, and saithe = orange) and the in-built ddPCR Poisson statistics.

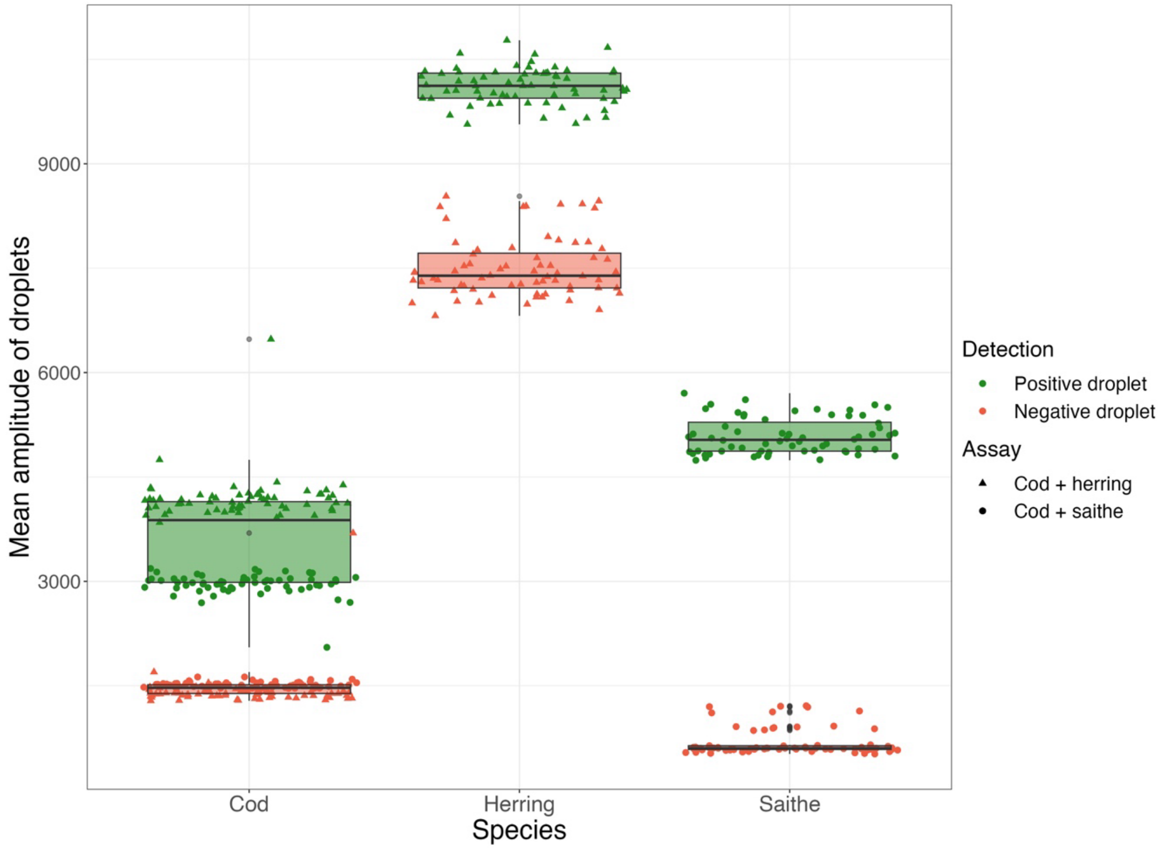

Figure S4. Mean amplitude of positive (green) and negative (red) droplet for all standard samples for two multiplexed assays (i) cod and herring (triangle symbol) and (ii) cod and saithe (circle symbol) indicating high amplitude (both negative and positive droplets) for herring target.

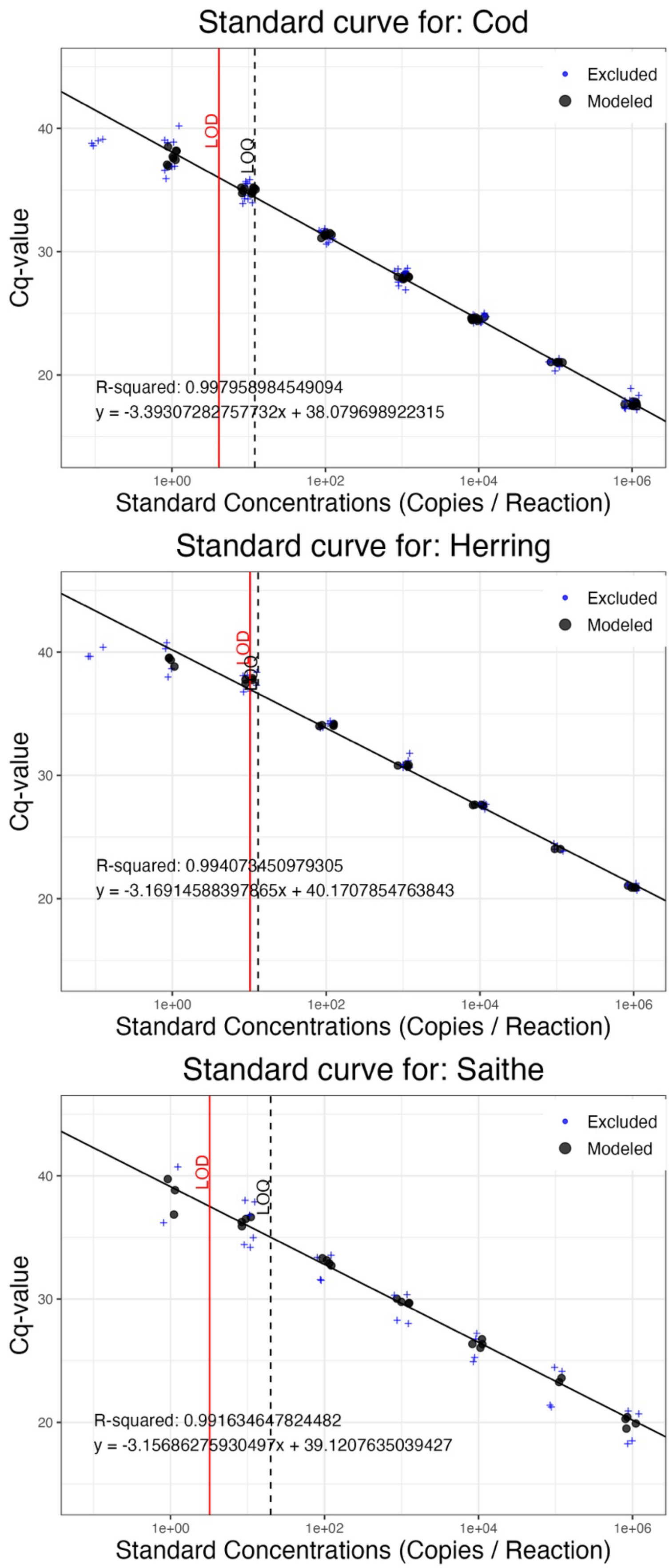

Figure S5. Limit of detection (LoD) and limit of quantification (LoQ) for all assays cod herring and saithe estimated as described in Klymus et al. (2020).

Table S1. Latent parameters estimate for three assays (cod, herring, and saithe) for both models (joint qPCR model and ddPCR model) implemented through Bayesian statistics (equation 1 through 5 and 8 through 9).

	Mean parameters estimates ± standard deviation					
	φ0	φ1	β0	β1	γ0	γ1
Cod	2.22 ± 0.19	1.35 ± 0.13	38.01 ± 0.12	-3.38 ± 0.02	0.28 ± 0.04	-0.28 ± 0.01
Herring	0.87 ± 0.20	2.09 ± 0.29	40.32 ± 0.13	-3.23 ± 0.02	0.43 ± 0.05	-0.44 ± 0.02
Saithe	0.42 ± 0.20	1.31 ± 0.24	38.58 ± 0.24	-3.09 ± 0.05	0.36 ± 0.05	-0.13 ± 0.02

	κ0	κ1
Cod	-7.05 ± 0.01	2.30 ± 0.00
Herring	-7.52 ± 0.02	2.24 ± 0.00
Saithe	-6.99 ± 0.02	2.26 ± 0.01

Table S2. Standard samples for three assays (cod, herring, and saithe) and the number of technical replicates run through qPCR ddPCR for each nominal concentration (expressed in log_{10}).

Assay	Log_{10} DNA concentration	Number of qPCR replicates	Number of ddPCR replicates
Cod	-3	-	16
Cod	-2	-	16
Cod	-1	14	16
Cod	0	22	16
Cod	1	20	16
Cod	2	17	16
Cod	3	18	16
Cod	4	18	16
Cod	5	11	-
Cod	6	18	-
Herring	-3	-	8
Herring	-2	-	Not included (outliers)
Herring	-1	8	8
Herring	0	12	8
Herring	1	10	8
Herring	2	9	8
Herring	3	10	8
Herring	4	10	8
Herring	5	6	-
Herring	6	10	-
Saithe	-3	-	8
Saithe	-2	-	8
Saithe	-1	6	8
Saithe	0	10	8
Saithe	1	10	8
Saithe	2	8	8
Saithe	3	8	8
Saithe	4	8	8
Saithe	5	6	8
Saithe	6	8	7

References:

Klymus, K. E., Merkes, C. M., Allison, M. J., Goldberg, C. S., Helbing, C. C., Hunter, M. E., Jackson, C. A., Lance, R. F., Mangan, A. M., Monroe, E. M., Piaggio, A. J., Stokdyk, J. P., Wilson, C. C., & Richter, C. A. (2020). Reporting the limits of detection and quantification for environmental DNA assays. *Environmental DNA*, *2*(3), 271–282. https://doi.org/10.1002/edn3.29
